# Supplementary figures and images for: Metagenomic analysis for taxonomic and functional potential of Polyaromatic hydrocarbons (PAHs) and Polychlorinated biphenyl (PCB) degrading bacterial communities in steel industrial soil
Source: PLoS One. 2022 Apr 29;17(4):e0266808. doi: 10.1371/journal.pone.0266808 (PMC9053811; doi:10.1371/journal.pone.0266808)

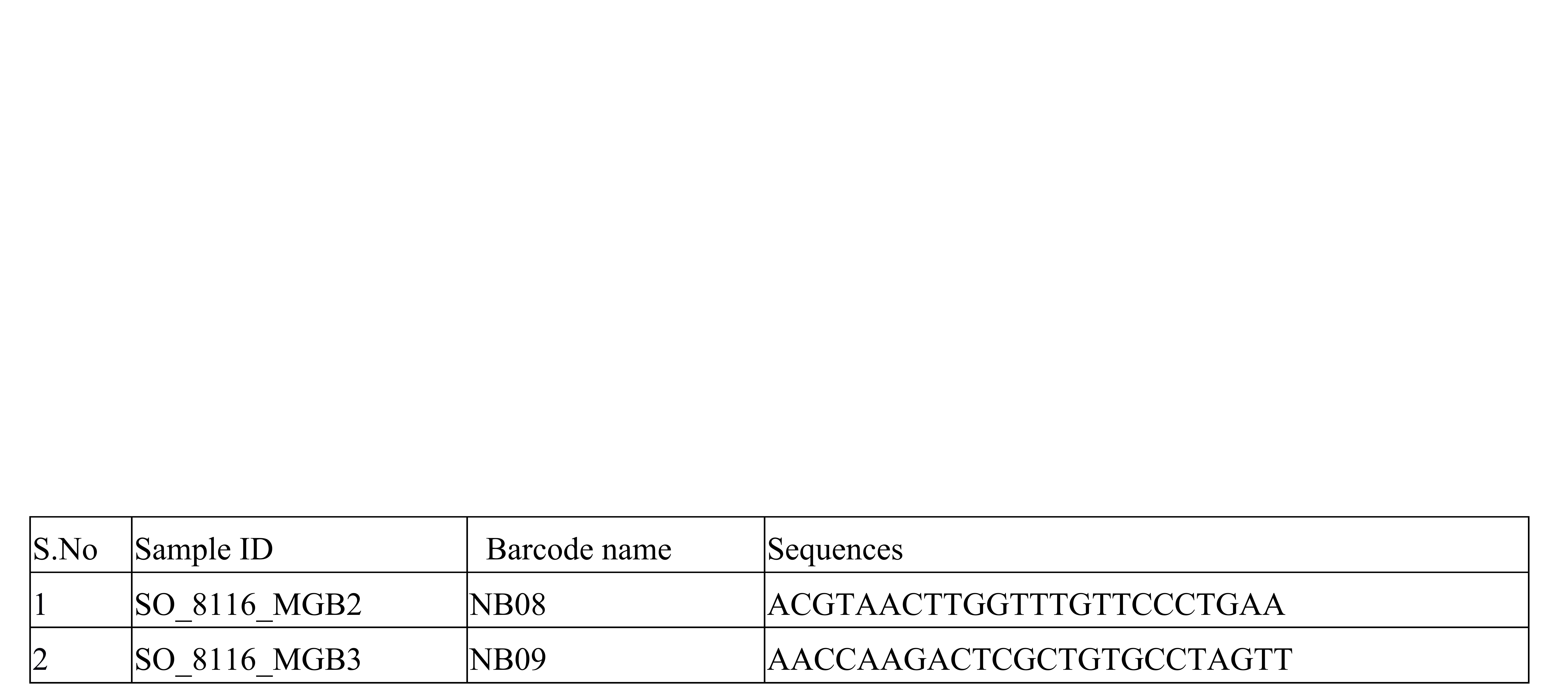

Supplement: S1 Table — (JPG) [file pone.0266808.s001.jpg]

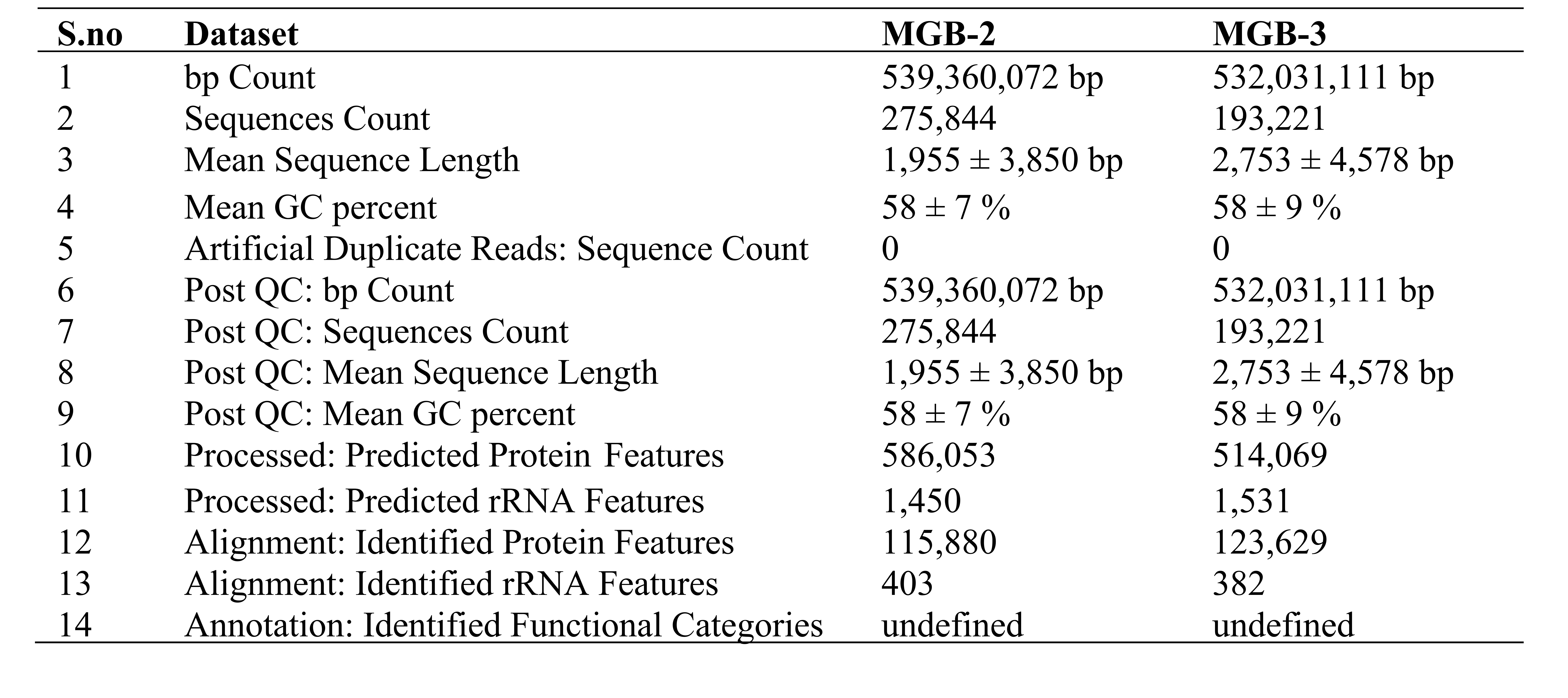

Supplement: S2 Table — (JPG) [file pone.0266808.s002.jpg]

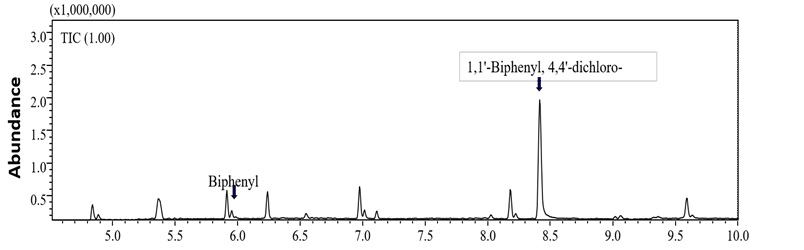

Supplement: S1 Fig — (JPG) [file pone.0266808.s003.jpg]

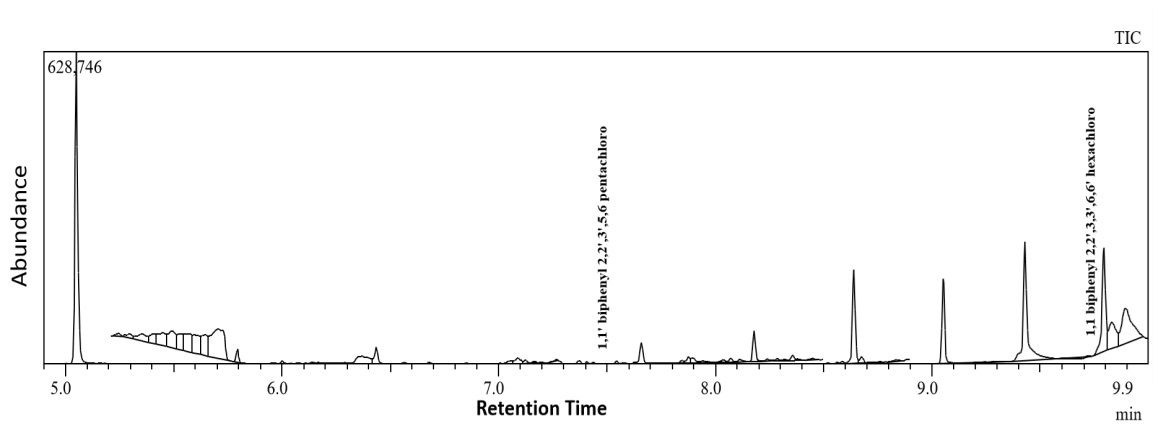

Supplement: S2 Fig — (JPG) [file pone.0266808.s004.jpg]

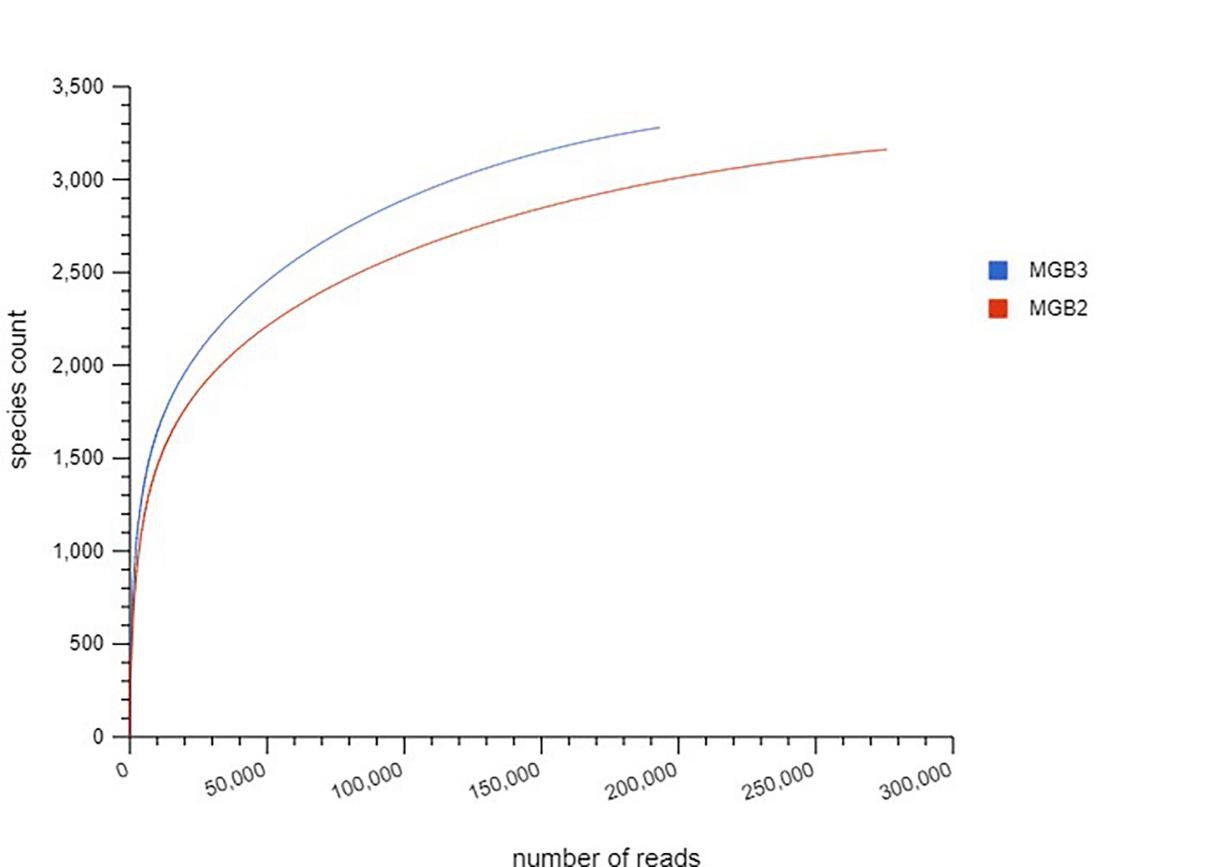

Supplement: S3 Fig — (JPG) [file pone.0266808.s005.jpg]

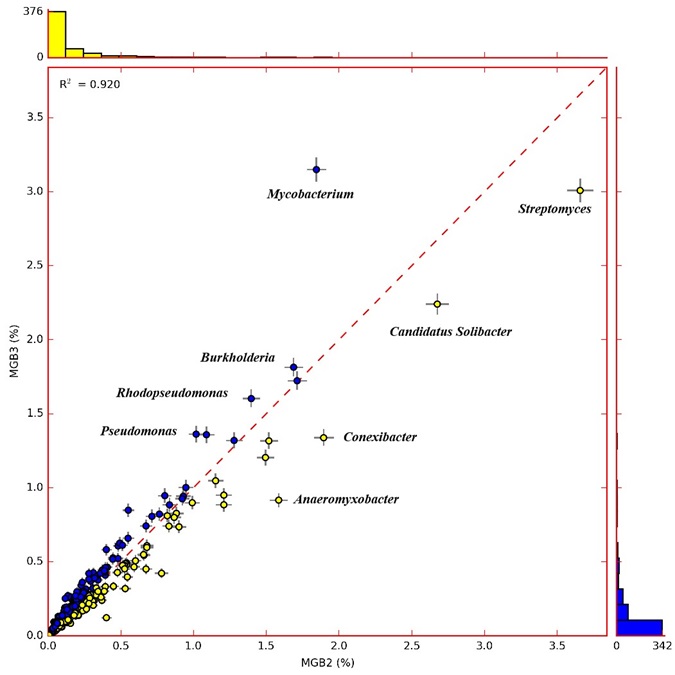

Supplement: S4 Fig — (JPG) [file pone.0266808.s006.jpg]

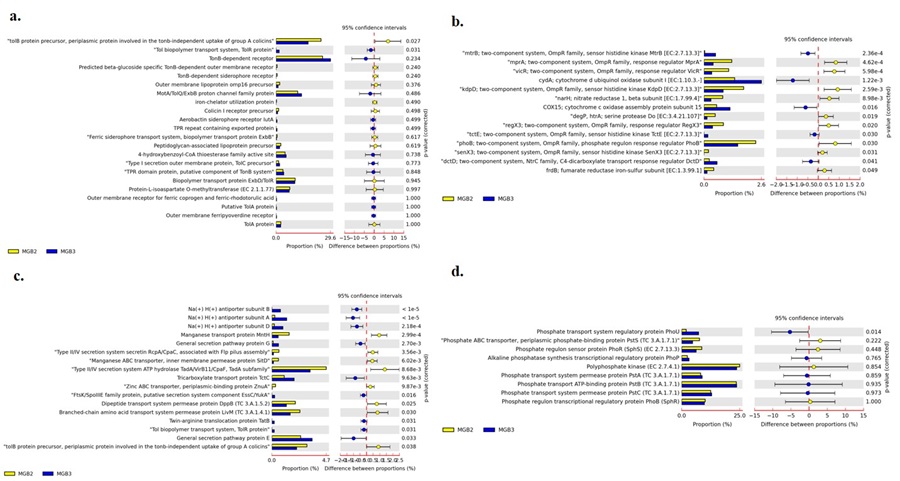

Supplement: S5 Fig — Comparison between MGB-2 and MGB-3 of functional gene annotation using STAMP using SEED subsystem a.) Tol and Ton b.) two-component system c.) membrane transport d.) Phosphate transporter. (JPG) [file pone.0266808.s007.jpg]

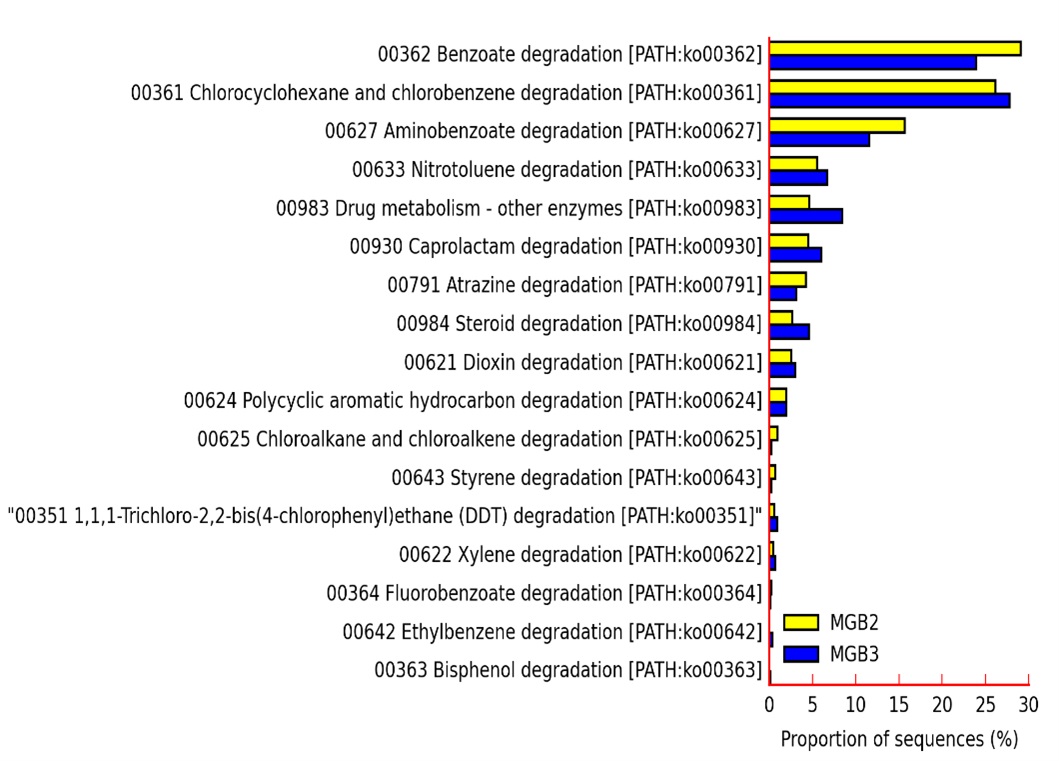

Supplement: S6 Fig — (JPG) [file pone.0266808.s008.jpg]

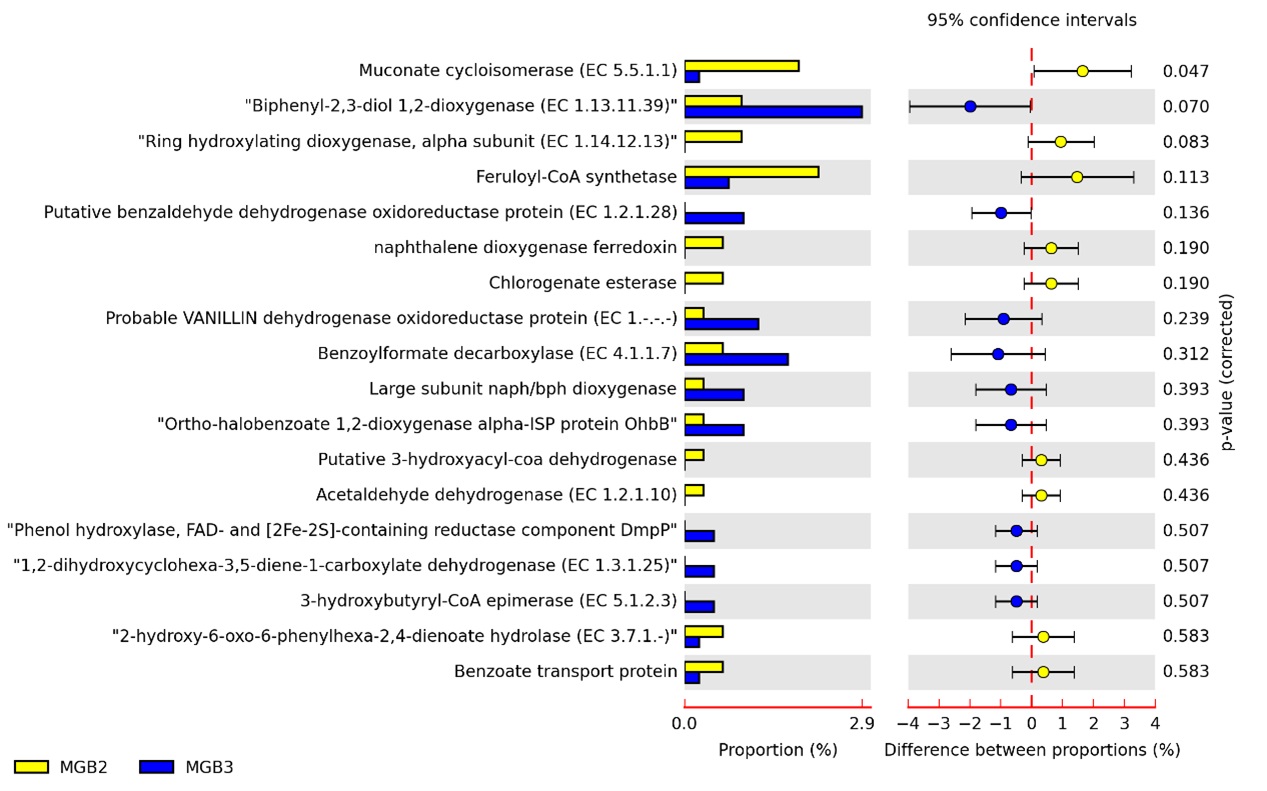

Supplement: S7 Fig — (JPG) [file pone.0266808.s009.jpg]

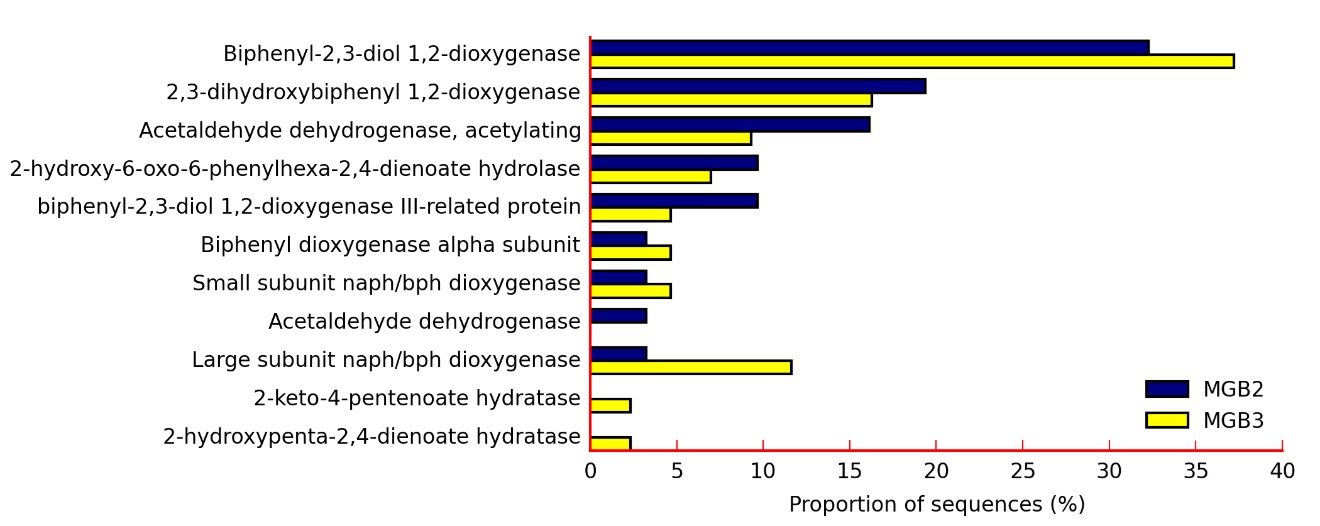

Supplement: S8 Fig — (JPG) [file pone.0266808.s010.jpg]

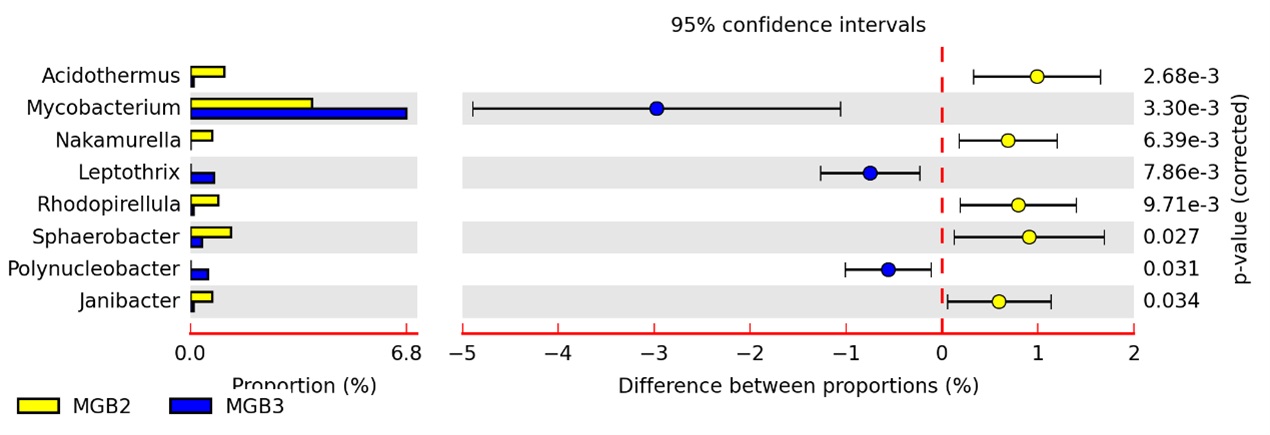

Supplement: S9 Fig — (JPG) [file pone.0266808.s011.jpg]
