# Supplementary material for: Metagenomic analysis for taxonomic and functional potential of Polyaromatic hydrocarbons (PAHs) and Polychlorinated biphenyl (PCB) degrading bacterial communities in steel industrial soil
Source: PLoS One. 2022 Apr 29;17(4):e0266808. doi: 10.1371/journal.pone.0266808 (PMC9053811; doi:10.1371/journal.pone.0266808)
Supplement: S1 File — (DOCX) [file pone.0266808.s012.docx]

# Supplementary sheet

# Metagenomic analysis for taxonomic and functional potential of Polyaromatic hydrocarbons (PAHs) and Polychlorinated biphenyl (PCB) degrading bacterial communities in steel industrial soil

Monika Sandhu*^a^*, Atish T. Paul*^b^* and Prabhat N. Jha*^a*^*

*^a^Department of Biological Sciences, Birla Institute of Technology and Science (BITS), Pilani, Pilani*

*^b^ Department of Pharmacy, Birla Institute of Technology and Science (BITS), Pilani Rajasthan, India*

**Table S1** Barcodes used for sequencing

| S.No | Sample ID | Barcode name | Sequences |
| --- | --- | --- | --- |
| 1 | SO_8116_MGB2 | NB08 | ACGTAACTTGGTTTGTTCCCTGAA |
| 2 | SO_8116_MGB3 | NB09 | AACCAAGACTCGCTGTGCCTAGTT |

**Table S2** The total number of unassembled and assembled reads used for downstream analyses

| **S.no** | **Dataset** | **MGB-2** | **MGB-3** |
| --- | --- | --- | --- |
| 1 | bp Count | 539,360,072 bp | 532,031,111 bp |
| 2 | Sequences Count | 275,844 | 193,221 |
| 3 | Mean Sequence Length | 1,955 ± 3,850 bp | 2,753 ± 4,578 bp |
| 4 | Mean GC percent | 58 ± 7 % | 58 ± 9 % |
| 5 | Artificial Duplicate Reads: Sequence Count | 0 | 0 |
| 6 | Post QC: bp Count | 539,360,072 bp | 532,031,111 bp |
| 7 | Post QC: Sequences Count | 275,844 | 193,221 |
| 8 | Post QC: Mean Sequence Length | 1,955 ± 3,850 bp | 2,753 ± 4,578 bp |
| 9 | Post QC: Mean GC percent | 58 ± 7 % | 58 ± 9 % |
| 10 | Processed: Predicted Protein Features | 586,053 | 514,069 |
| 11 | Processed: Predicted rRNA Features | 1,450 | 1,531 |
| 12 | Alignment: Identified Protein Features | 115,880 | 123,629 |
| 13 | Alignment: Identified rRNA Features | 403 | 382 |
| 14 | Annotation: Identified Functional Categories | undefined | undefined |

**
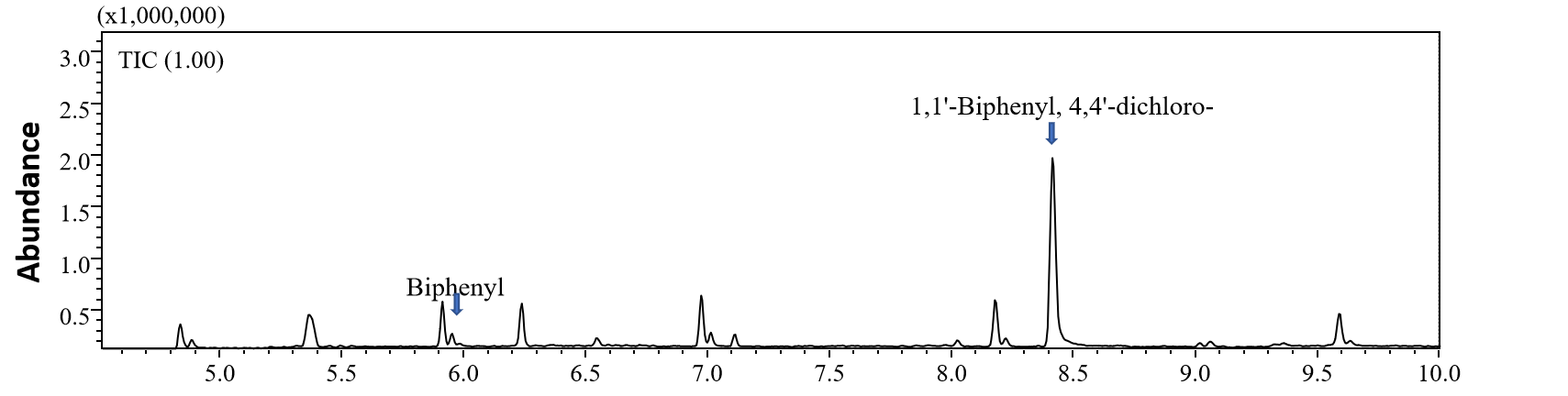
**

**S1 Fig.** **PCB identified in MGB-2 sample**

**
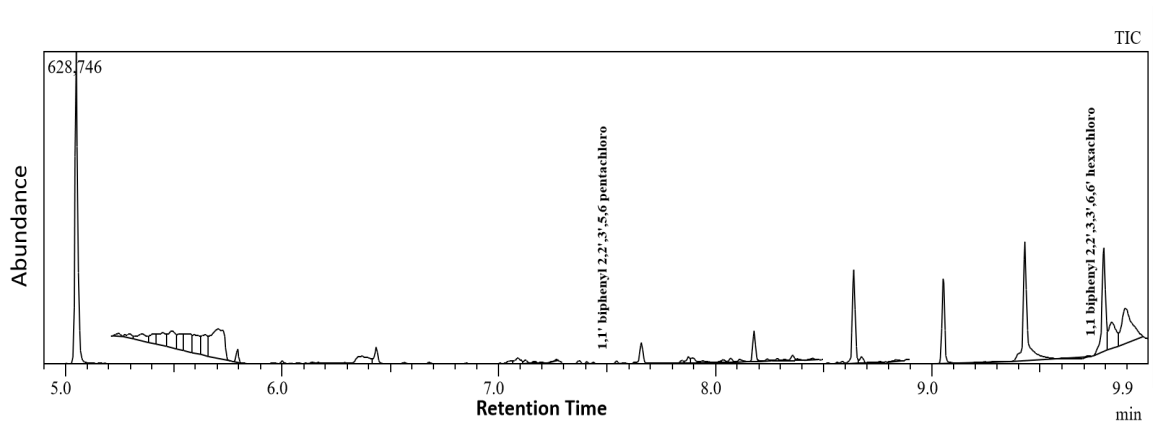
**

**S2 Fig.** **PCB identified in MGB-3 sample**


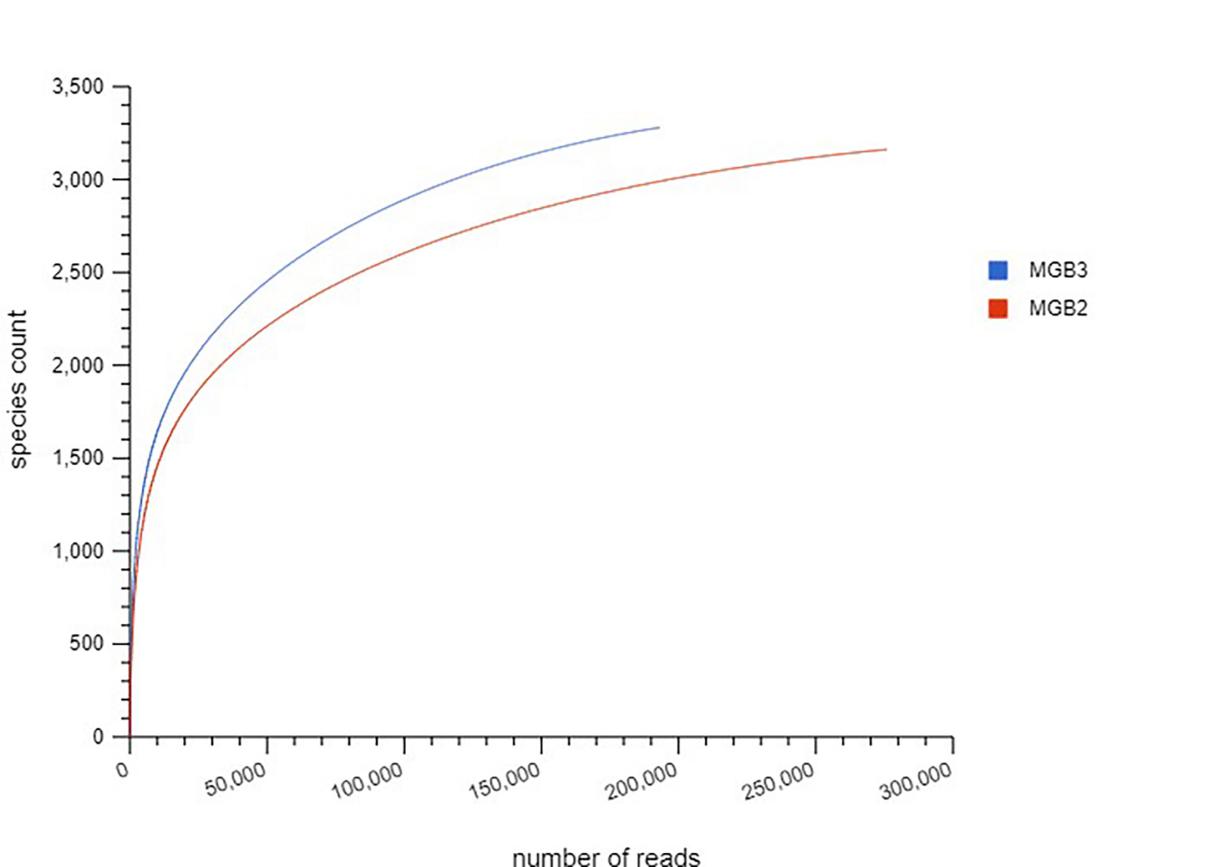


**S3 Fig**. Rarefaction curve represented species richness indicating function of number of reads (y-axis) per MGB-2 and MGB-3 (x- axis)


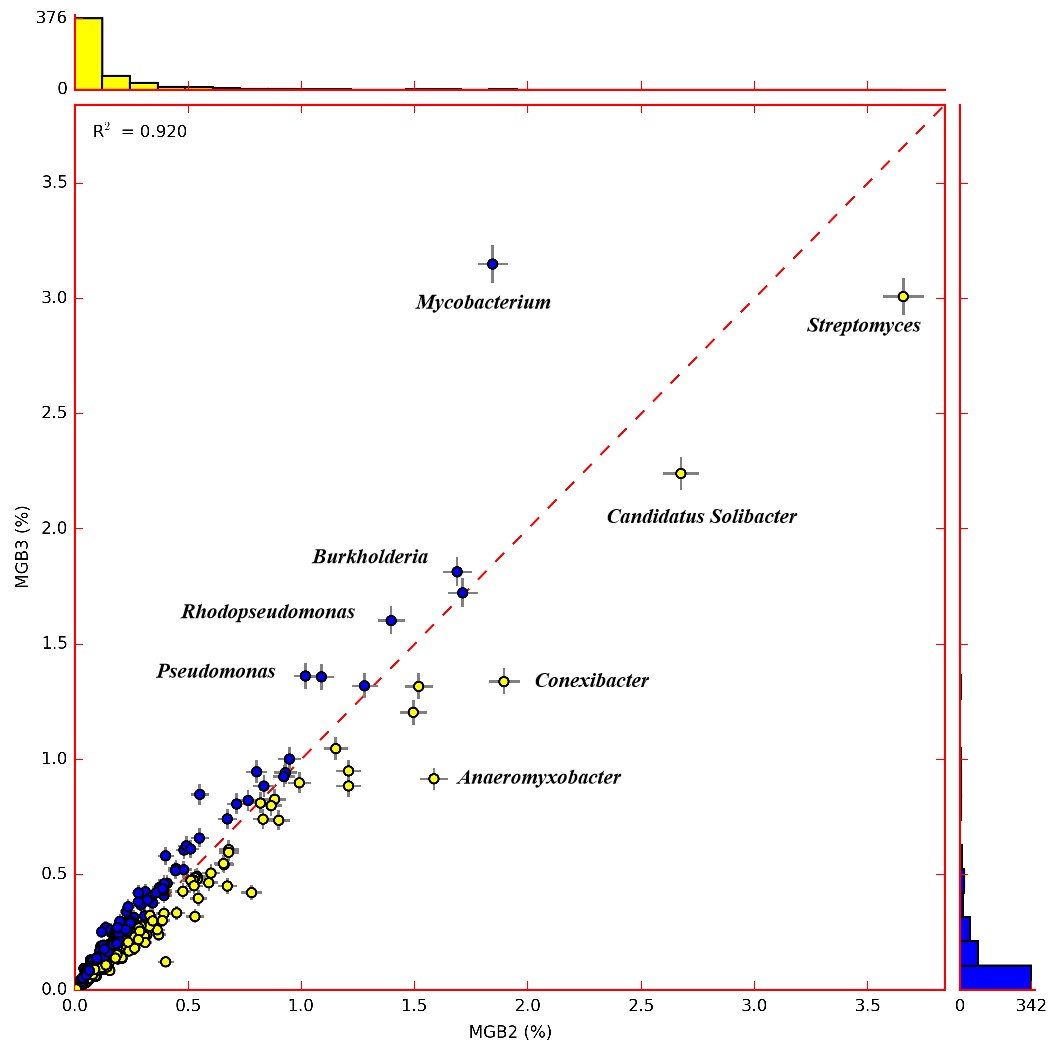


S4 Fig. The scatter plot indicated a linear relationship between MGB-2 and MGB-3

**
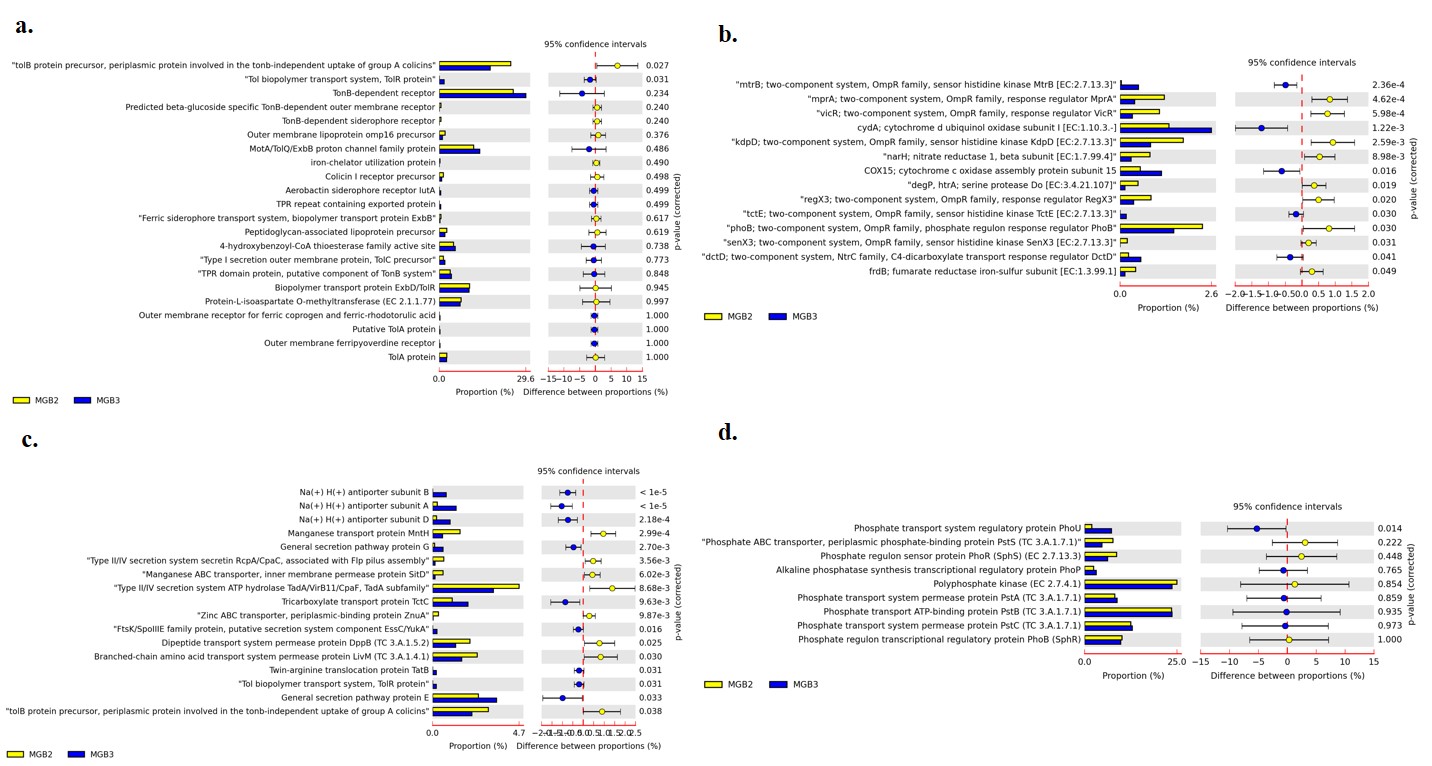
 S5 Fig.** Comparison between MGB-2 and MGB-3 of functional gene annotation using STAMP using SEED subsystem a.) Tol and Ton b.) two-component system c.) membrane transport d.) Phosphate transporter


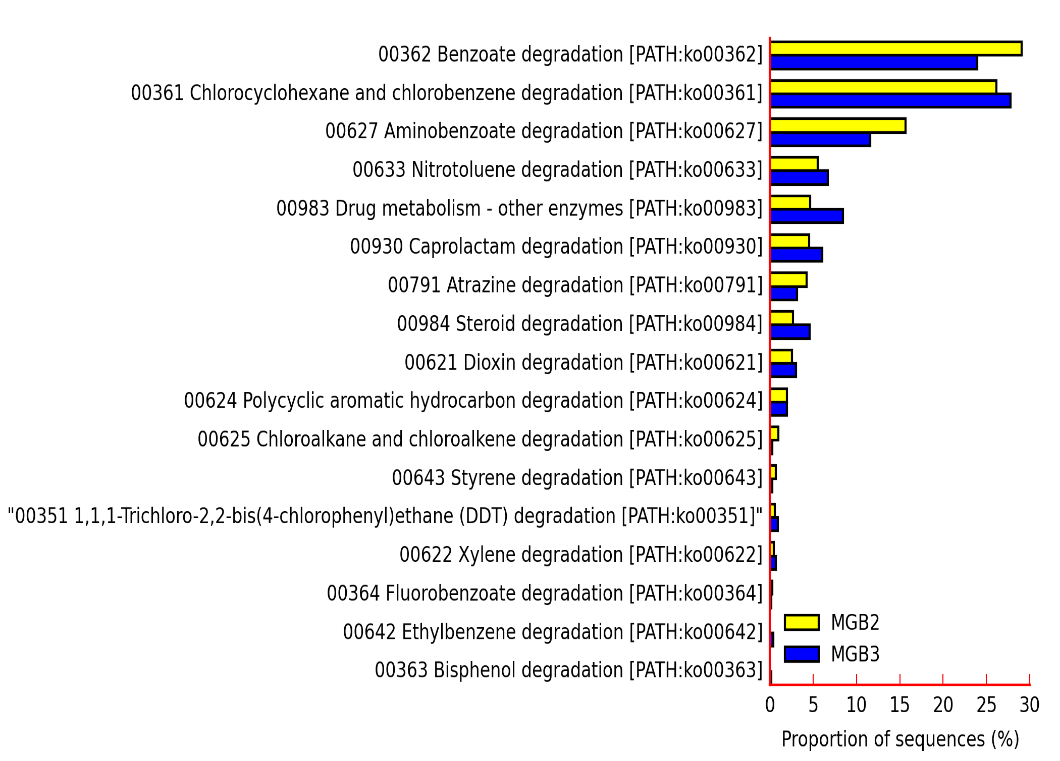


**S6 Fig.** Annotated pathways for xenobiotic degradation and metabolism accounted for metagenome MGB-2 and MGB-3


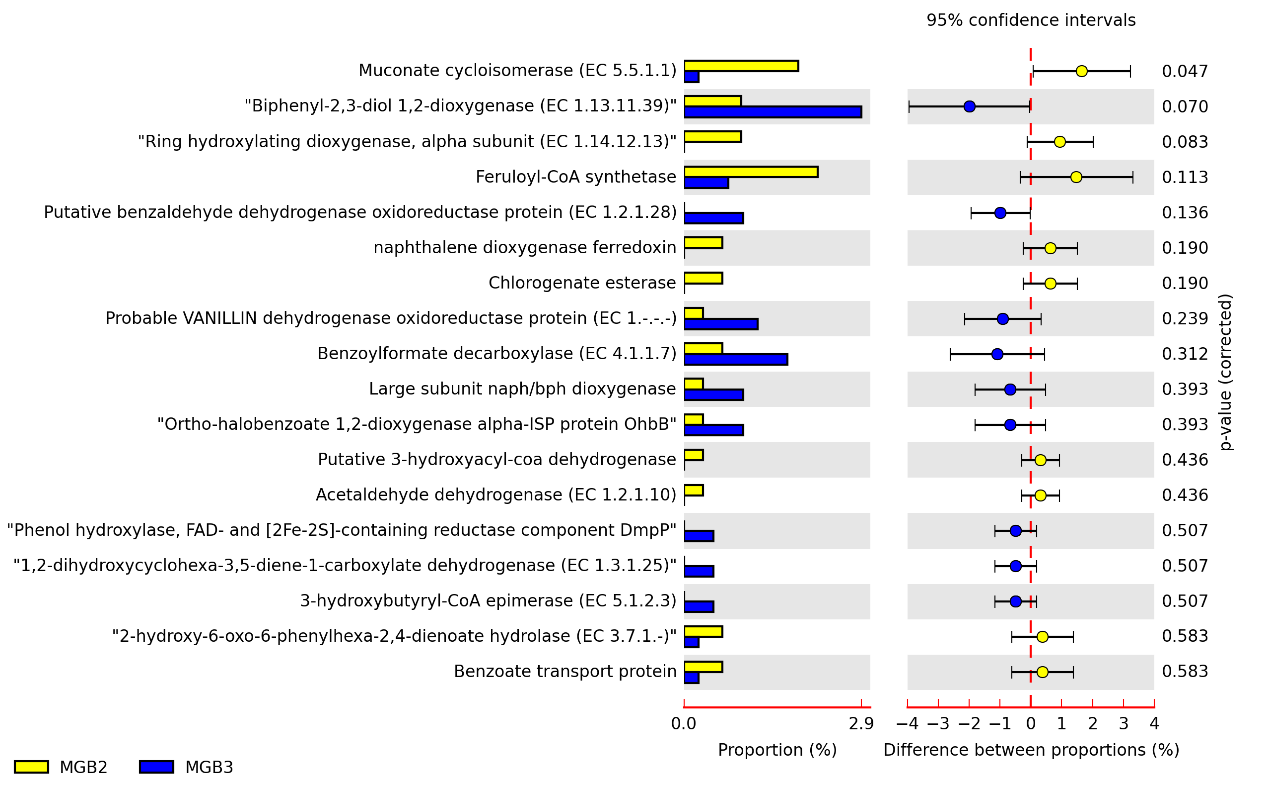


**S7 Fig****.** Comparative analysis of metagenome MGB-2 and MGB-3 based on Peripheral aromatic degradation metabolism at SEED subsystem level by using STAMP


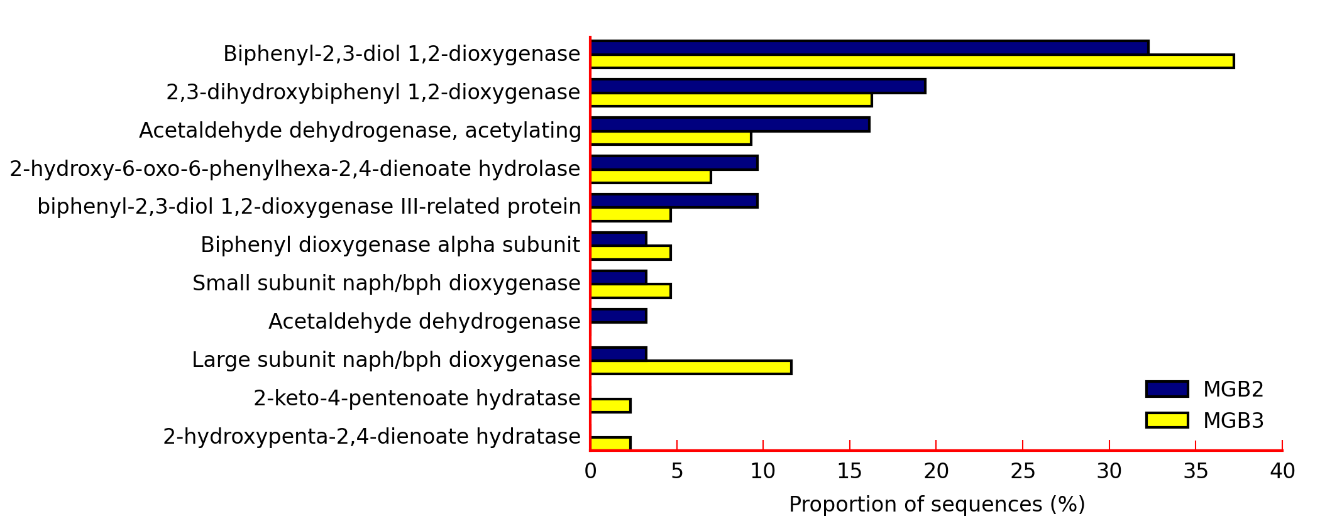


**S8 Fig.** Comparative analysis of metagenome MGB-2 and MGB-3 based on biphenyl degradation metabolism SEED level by using STAMP


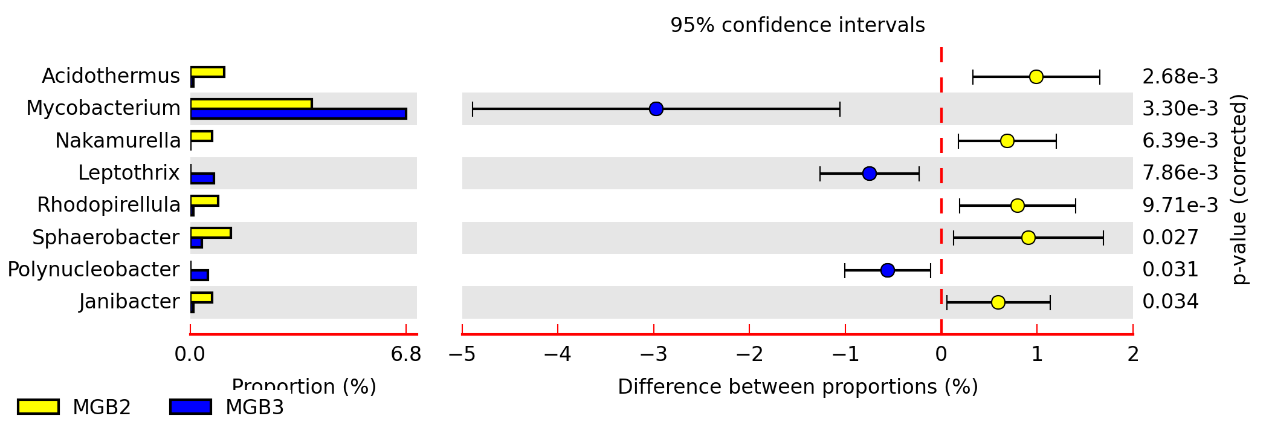
 **S9Fig.** Comparative analysis of metagenome MGB-2 and MGB-3 based on aromatic degradation metabolism at ReFSeq genus level by using STAMP
