# Supplementary material for: Metagenomic analysis for taxonomic and functional potential of Polyaromatic hydrocarbons (PAHs) and Polychlorinated biphenyl (PCB) degrading bacterial communities in steel industrial soil
Source: PLoS One. 2022 Apr 29;17(4):e0266808. doi: 10.1371/journal.pone.0266808 (PMC9053811; doi:10.1371/journal.pone.0266808)
Supplement: Attachment — Submitted filename: Reviewer document-PONE-D-21-32138.docx [file pone.0266808.s013.docx]

**Manuscript Number:	PONE-D-21-32138**

**Full Title:	Metagenomic analysis for taxonomic and functional potential of Polyaromatic hydrocarbons (PAHs) and Polychlorinated biphenyl (PCB) degrading bacterial communities in steel industrial soil**

**Authors:	Monika Sandhu, Atish T. Paul, and Prabhat N. Jha**

# **General Comments**

Polyaromatic hydrocarbons and polychlorinated biphenyls have been discovered in various industrial soils globally. This has led to the intensive identification and characterization of degrading bacterial species or communities. Sandhu et al explored the identification and characterization of bacterial communities isolated from a steel industrial-contaminated soil. Whole-genome metagenomic analysis, as well as pathway and taxonomic *vis-à-vis* functional analysis identification, was undertaken to identify the bacterial PCB and PAH degraders amongst the community members. As an exhaustive community identification process, it gives a unique outlook on PAH and PCB degradation in steel industry-contaminated site, and, therefore, I would like to recommend this work for acceptance by the *Plos-ONE* only after the minor issues highlighted below are addressed.

# **Minor comments**

The red highlighted areas show the edited problem area OR errors in the line(s) quoted in the original manuscript

## ***Keywords Section***

Kindly edit the keyword highlighted as follows below;

persistent organic pollutant,

## ***Introduction***

Lines 48-50: Please amend the sentence as shown below;

Persistent organic pollutants (POPs) are anthropogenic chemicals that are enlisted as priority environmental pollutants due to their toxicity and persistence in the environment for a prolonged period [1].

Lines 76-77: Kindly revise the sentence as follows;

However, most of these studies are based on the 16S rRNA gene sequence, which does not highlight the metabolic potential of resident microorganisms.

Lines 84-88: Please modify the sentence as shown below

Therefore, the present study aimed at investigating and providing an insight into the diversity of the bacterial community, metabolic potential of the dominant bacterial community in the contaminated soil collected from nearby regions of Bhilai steel plant (one of Asia’s biggest steel plants) in Chhattisgarh, India, and to correlate their functional characteristics to the biodegradation pathways.

## ***Materials and Methods***

Lines 105-106: Kindly edit the sentence as shown below;

After allowing it to stand for 30 min, 10 ml of acetone and hexane (1:1; v/v) were added to the falcon and vortexed for 3 min.

Lines 132-133: Please amend the sentence as shown below;

Adapter ligation (BAM), cleaning of library mix, and elution of sequencing library was done as per Kumar et al. [13] and was further used for whole-genome sequencing.

## ***Results and Discussion***

Lines 178-180: Kindly revise the sentence as shown below;

Several studies have confirmed that the soil with high microbial diversity has high P and low C/P ratios, while the environment with less P and high C/P ratios shows a low microbial diversity [19].

Lines 204-205: Please edit the sentence as shown below;

Hence it was deemed fit to estimate the level of POPs, mainly PAHs and PCBs, in given soil samples by GC-MS/MS using Scan/SIM mode.

Lines 222-225: Kindly amend the sentence as shown below;

The present results corroborate with previous studies conducted on the Bhilai steel plant (Raipur, Chhattisgarh) soil, reporting the presence of PCBs ranging from dichlorinated to hexachlorinated biphenyl in the sludge [28].

Lines 232-234: Please modify the sentence as shown below;

Whole-genome metagenomics studies are used not only to study the taxonomic diversity but also to elucidate the metabolic pathways required for understanding pollutant degradation [29].

Lines 261-266: Kindly amend the sentence as shown below;

Both samples included all six classes of Proteobacteria namely *Alphaproteobacteria* (18.6%; 22.4%), *Betaproteobacteria* (8.8%; 10.7%), *Deltaproteobacteria* (8.2%; 7.1%), *Epsilonproteobacteria*	(0.3%;	0.3%),	*Gammaproteobacteria* (7.2%; 8.9%) and *Zetaproteobacteria* (0.04%; 0.03%) with difference in their relative abundance, which indicates the presence of diverse members playing a vital role in photosynthesis, nitrogen fixation, sulfur, and phosphorus metabolism [31].

Lines 269-270: Please revise the sentence as shown below;

The analysis showed close similarity in both metagenomes; however, they differ in relative abundance.

Lines 286-288: Kindly edit the sentence as shown below;

Similarly, members of *Mycobacterium*, *Bradyrhizobium*, *Burkholderia* [34-35], and *Pseudomonas* [36-37] are well-known aromatic pollutant degraders in contaminated soils [38].

Lines 289-291: Please revise the sentence as shown below;

Within the metagenome of MGB-2 and MGB-3, Archaeal communities showed a dominance of *Euryarchaeota* (76.7%; 81.3%) at phylum level, followed by *Crenarchaeota* (13.7%; 13.2%) *Thaumarchaeota* (8.7%; 4.8%) and *Korarchaeota* (0.9%; 0.7%).

Lines 296-298: Please amend the sentence as shown below;

However, a two-way statistical comparison of Archaea at genus level revealed *Nitrosopumilus* (p = 1.24 e-4) and *Cenarchaeum* (p = 5.18 e-3) in MGB-2 while *Methanobrevibacterium* (p = 0.012) in MGB-3 to predominate, indicating more methanogenesis occurring in MGB-3 [39].

Lines 302-303: Kindly modify the sentence as shown below;

Relative abundances of Archaeal communities were more in MGB-2, which was associated with the higher soil moisture and POPs content.

Lines 334-336: Please edit the sentence as shown below;

The availability number and abundance of reads for phosphate mechanism in low phosphate-phosphorous environments indicated these mechanisms that help them cope within such environments.

Lines 343-345: Kindly revise the sentence as shown below;

MGB-3 showed higher Na+/H+ transporters that/which indicate the exchange of the ions across the membrane to maintain homeostasis.

Lines 352-354: Please amend the sentence as shown below;

The presence of reads for biphenyl degradation, dioxin degradation, PAH degradation pathways can be further correlated with the presence of PCBs and PAHs, as detected in the MGB-2 and MGB-3 samples (Fig 5B).

Lines 364-366: Kindly modify the sentence as shown below;

The presence of the above-mentioned enzymes suggests that the degradation of chlorobenzene is catalyzed via the ortho-cleavage pathway in two communities [42], and the meta-cleavage pathway [43].

Lines 367-369: Please revise the sentence as shown below;

The degradation pathway mainly of PAHs and PCBs is highlighted in the present study as the collected soil samples MGB-2 and MGB-3 were found to be highly contaminated with these organic pollutants.

Lines 370-373: Kindly edit the sentence as shown below;

The presence of genes encoding hydroxychromene-2-carboxylate isomerase (*nahD*), naphthalene dioxygenase (*nahAc*), salicylate hydroxylase (*nahG*), and other genes including *nidA*, *nidB*, *nidD*, *phdF*, *phdG*, *phdI*, and *phdJ* indicated the presence of a complete pathway of PAH degradation.

Lines 384-386: Please amend the sentence as shown below;

The annotated reads for various genes, including *bphA*, *bphC*, *bphD*, *bphE*, and *bphF*, corresponding to the biphenyl degradation pathway, were identified in both samples in Table 4b.

Lines 388-389: Kindly modify the sentence as follows;

Biphenyl 2, 3-diol, 1, 2-dioxygenase (*bphC*) is another important enzyme that was noted in both metagenomes.

Lines 405-409: Please edit the sentences as shown below;

These enzymes are contributed possibly by members of genera *Aromatoleum*, *Arthrobacter*, *Burkholderia*, *Bradyrhizobium*, *Cupriavidus*, *Magnetospirullum*, Methylobacterium, Rhodomicrobium, *Rhodopseudomonas*, and *Polaromonas* in both metagenome communities, suggesting a synergistic degradation of benzoate.

Lines 413-419: Kindly revise the sentences as follows;

On the other hand, genes encoding biphenyl 2,3 diol dioxygenase, benzaldehyde dehydrogenase, nap/bph dioxygenase, ortho-halobenzoate 1,2-dioxygenase, phenol dioxygenase, and 1,2-dihydroxycyclohexa-3,5-diene-1-carboxylate dehydrogenate were abundant in MGB-3 (Fig S5). Similarly, a two-way comparison between MGB-2 and MGB-3 of functional gene annotation using SEED subsystem for aromatic degradation revealed a significant level of abundance for benzoate ligase (p = 2.63 e-3) and 4-hydroxyphenylacetate 3-monooxygenase (p = 8.93 e-3) in MGB-2.

Lines 423-426: Please amend the sentences as shown below;

Furthermore, a comparison between MGB-2 and MGB-3 at functional gene annotation using RefSeq for aromatic degradation revealed a significant level of abundance of *Acidothermus* (p=2.68 e-3), *Nakamurella* (p=6.39 e-3) in MGB-2 while *Mycobacterium* (p = 3.30 e-3), *Leptothrix* (p=7.89 e-3) and *Polynuclearbacter* (p=0.034) in MGB-3 (Fig. S9).

Lines 430-432: Kindly modify the sentences as observed below;

Therefore, the reconstructions of complete biphenyl/PCB and PAH degradative pathways, based on the annotated genes, were done and shown in Fig 6. Cytoscape-based networking revealed microbial interaction in the xenobiotic biodegradation pathway.

## ***Figure Captions***

Lines 665-667: Please edit the caption as observed below;

Fig 3 (A). Taxonomic analysis beta diversity of MGB-2 and MGB-3 on Bary -Curtis dissimilarity. (B) Krona plot demonstrated the relative abundance of bacterial taxa across phylum to genus level hierarchy of a. MGB-2; b. MGB-3

## ***Supplementary Information Document***

Kindly edit the word “Supplementary” in all areas mentioned incorrectly throughout the Supplementary Information document.

Kindly amend the quoted caption as seen below;

Supplementary Fig S3. Rarefaction curve represented species richness indicating function of number of reads (y-axis) per MGB-2 and MGB-3 (x-axis)

Please edit the quoted caption as follows;

Supplementary Fig S5. Comparison between MGB-2 and MGB-3 of functional gene annotation using STAMP using SEED subsystem a.) Tol and Ton b.) two-component system c.) membrane transport d.) Phosphate transporter
