# Supplementary material for: Metagenomic analysis for taxonomic and functional potential of Polyaromatic hydrocarbons (PAHs) and Polychlorinated biphenyl (PCB) degrading bacterial communities in steel industrial soil
Source: PLoS One. 2022 Apr 29;17(4):e0266808. doi: 10.1371/journal.pone.0266808 (PMC9053811; doi:10.1371/journal.pone.0266808)
Supplement: Attachment — Submitted filename: Final Response to reviewers comment.docx [file pone.0266808.s014.docx]

| Editor’s comment: **Editor’s comment:** \| **Response to editors’ comment**; We require you to either (1) present written permission from the copyright holder to publish these figures specifically under the CC BY 4.0 license, or (2) remove the figures from your submission: \| Figure 1 has been removed from the manuscript and manuscript have been updated accordingly Reviewer #1: **Reviewer #1 comment** \| **Response**; Why did the authors choose to study the PAH and PCB degrading genes in soil collected from steel industry? What is the scientific relevance of a steel plant? Why not soil/sludge from petrochemical refining units? \| There are several reports indicating that hazardous pollutants PAHs and PCBs are released by various industrial activities such as iron and steel plants (Aydin et al., 2014; Khaparde et al. 2016; Yang et al., 2002). These pollutants are emitted from iron and steel production, like coke production, sintering, iron production, casting, and cooling, and steel manufacturing. As Bhilai steel plant is India’s first and major steel plant, the current work focuses on the microbial community of the Steel plant.; What is the scientific evidence that the communities identified have PAH/PCB degradation potential? There is none mentioned/presented in this study. Presence of a PAH degrading gene doesn't confirm its expression. \| There are several studies that had revealed the importance of metagenomic analysis of PAH/PCB contaminated soil in exploring the potential degrading bacterial community and have exploited potential candidate for further bioremediation of such pollutants. (Zafra et al., 2016; Czarny et al 2017) Current work aims to exploring the potential of microbial flora and respective PAH/ PCB degrading pathways based on metagenome only. We do agree that the mere presence of genes does not confirm the expression of gene which is a subject of future analysis.; Presence of a pollutant in soil doesn't confirm its degradation by the microbial communities present there? The growth dynamics. enzyme activity and gene expression in addition to PAH/PCB degradation can only confirm it. The idea lacks scientific evidence. \| The present study aimed to understand the structure and composition of microbial communities present in PCB/PAH contaminated soil near the steel industry and their genetic potential to degrade them employing cultivation-independent whole genome-metagenome analysis. The work provides insights regarding the microbial populations and metabolic pathways which can be required for complete degradation of the above pollutant by a given community. The estimation of microbial activity, enzymes, and gene expression studies is the subject of independent studies which are being carried out in our laboratory. Reviewer #2: This manuscript presents an exhaustive community identification process that gives a unique outlook on PAH and PCB degradation in steel industry-contaminated site. My recommendation for this work is acceptance only after the minor issues highlighted in the reviewer document are addressed. **Minor comments** The red highlighted areas show the edited problem area OR errors in the line(s) quoted in the original manuscript | | |
| --- | --- | --- |
| **Section** | **Reviewer’s comment** | **Response to Reviewers’** |
| **Keywords Section** | Kindly edit the keyword highlighted as follows below;  persistent organic pollutant, | Edited as per the reviewer comment |
| ***Introduction*** | | |
| Lines 48-50 | Please amend the sentence as shown below;  Persistent organic pollutants (POPs) are anthropogenic chemicals that are enlisted as priority environmental pollutants due to their toxicity and persistence in the environment for a prolonged period [1]. | Amended as per the reviewer comment |
| Lines 76-77 | Kindly revise the sentence as follows; However, most of these studies are based on the 16S rRNA gene sequence, which does not highlight the metabolic potential of resident microorganisms. | Revised as per the reviewer comment |
| Lines 84-88 | Please modify the sentence as shown below:  Therefore, the present study aimed at investigating and providing an insight into the diversity of the bacterial community, metabolic potential of the dominant bacterial community in the contaminated soil collected from nearby regions of Bhilai steel plant (one of Asia’s biggest steel plants) in Chhattisgarh, India, and to correlate their functional characteristics to the biodegradation pathways. | Modified as per the reviewer comment |
| ***Materials and Methods*** | | |
| Lines 105-106: | Kindly edit the sentence as shown below;  After allowing it to stand for 30 min, 10 ml of acetone and hexane (1:1; v/v) were added to the falcon and vortexed for 3 min. | Edited as per the reviewer comment |
| Lines 132-133: | Please amend the sentence as shown below;  Adapter ligation (BAM), cleaning of library mix, and elution of sequencing library was done as per Kumar et al. [13] and was further used for whole-genome sequencing. | Amended as per the reviewer comment |
| ***Results and Discussion*** | | |
| Lines 178-180: | Kindly revise the sentence as shown below; Several studies have confirmed that the soil with high microbial diversity has high P and low C/P ratios, while the environment with less P and high C/P ratios shows a low microbial diversity [19]. | Revised as per the reviewer comment |
| Lines 204-205: | Please edit the sentence as shown below;  Hence it was deemed fit to estimate the level of POPs, mainly PAHs and PCBs, in given soil samples by GC-MS/MS using Scan/SIM mode. | Edited as per reviewer comment |
| Lines 222-225: | Kindly amend the sentence as shown below;  The present results corroborate with previous studies conducted on the Bhilai steel plant (Raipur, Chhattisgarh) soil, reporting the presence of PCBs ranging from dichlorinated to hexachlorinated biphenyl in the sludge [28]. | Amended as per the reviewer comment |
| Lines 232-234: | Please modify the sentence as shown below;  Whole-genome metagenomics studies are used not only to study the taxonomic diversity but also to elucidate the metabolic pathways required for understanding pollutant degradation [29]. | Modified as per the reviewer comment |
| Lines 261-266: | Kindly amend the sentence as shown below;  Both samples included all six classes of Proteobacteria namely *Alphaproteobacteria* (18.6%; 22.4%), *Betaproteobacteria* (8.8%; 10.7%), *Deltaproteobacteria* (8.2%; 7.1%), *Epsilonproteobacteria* (0.3%; 0.3%), *Gammaproteobacteria* (7.2%; 8.9%) and *Zetaproteobacteria* (0.04%; 0.03%) with difference in their relative abundance, which indicates the presence of diverse members playing a vital role in photosynthesis, nitrogen fixation, sulfur, and phosphorus metabolism [31]. | Amended as per the reviewer comment |
| Lines 269-270: | Please revise the sentence as shown below;  The analysis showed close similarity in both metagenomes; however, they differ in relative abundance. | Revised as per reviewer comment |
| Lines 286-288: | Kindly edit the sentence as shown below;  Similarly, members of *Mycobacterium*, *Bradyrhizobium*, *Burkholderia* [34-35], and *Pseudomonas* [36-37] are well-known aromatic pollutant degraders in contaminated soils [38]. | Edited as per reviewer comment |
| Lines 289-291: | Please revise the sentence as shown below;  Within the metagenome of MGB-2 and MGB-3, Archaeal communities showed a dominance of *Euryarchaeota* (76.7%; 81.3%) at phylum level, followed by *Crenarchaeota* (13.7%; 13.2%) *Thaumarchaeota* (8.7%; 4.8%) and *Korarchaeota* (0.9%; 0.7%). | Revised as per reviewer comment |
| Lines 296-298: | Please amend the sentence as shown below;  However, a two-way statistical comparison of Archaea at genus level revealed *Nitrosopumilus* (p = 1.24 e-4) and *Cenarchaeum* (p = 5.18 e-3) in MGB-2 while *Methanobrevibacterium* (p = 0.012) in MGB-3 to predominate, indicating more methanogenesis occurring in MGB-3 [39]. | Amended as per reviewer comment |
| Lines 302-303: | Kindly modify the sentence as shown below;  Relative abundances of Archaeal communities were more in MGB-2, which was associated with the higher soil moisture and POPs content. | Modified as per reviewer comment |
| Lines 334-336: | Please edit the sentence as shown below;  The availability number and abundance of reads for phosphate mechanism in low phosphate-phosphorous environments indicated these mechanisms that help them cope within such environments. | Edited as per reviewer comment |
| Lines 343-345: | Kindly revise the sentence as shown below;  MGB-3 showed higher Na+/H+ transporters that/which indicate the exchange of the ions across the membrane to maintain homeostasis. | Revised as per reviewer comment |
| Lines 352-354: | Please amend the sentence as shown below;  The presence of reads for biphenyl degradation, dioxin degradation, PAH degradation pathways can be further correlated with the presence of PCBs and PAHs, as detected in the MGB-2 and MGB-3 samples (Fig 5B). | Amended as per reviewer comment |
| Lines 364-366: | Kindly modify the sentence as shown below;  The presence of the above-mentioned enzymes suggests that the degradation of chlorobenzene is catalyzed via the ortho-cleavage pathway in two communities [42], and the meta- cleavage pathway [43]. | Modified as per reviewer comment |
| Lines 367-369: | Please revise the sentence as shown below;  The degradation pathway mainly of PAHs and PCBs is highlighted in the present study as the collected soil samples MGB-2 and MGB-3 were found to be highly contaminated with these organic pollutants. | Revised as per reviewer comment |
| Lines 370-373: | Kindly edit the sentence as shown below; The presence of genes encoding hydroxychromene-2-carboxylate isomerase (*nahD*), naphthalene dioxygenase (*nahAc*), salicylate hydroxylase (*nahG*), and other genes including *nidA*, *nidB*, *nidD*, *phdF*, *phdG*, *phdI*, and *phdJ* indicated the presence of a complete pathway of PAH degradation. | Edited as per reviewer comment |
| Lines 384-386: | Please amend the sentence as shown below;  The annotated reads for various genes, including *bphA*, *bphC*, *bphD*, *bphE*, and *bphF*, corresponding to the biphenyl degradation pathway, were identified in both samples in Table 4b. | Amended as per reviewer comment |
| Lines 388-389: | Kindly modify the sentence as follows;  Biphenyl 2, 3-diol, 1, 2-dioxygenase (*bphC*) is another important enzyme that was noted in both metagenomes. | Modified as per reviewer comment |
| Lines 405-409: | Please edit the sentences as shown below;  These enzymes are contributed possibly by members of genera *Aromatoleum*, *Arthrobacter*, *Burkholderia*, *Bradyrhizobium*, *Cupriavidus*, *Magnetospirullum*, Methylobacterium, Rhodomicrobium, *Rhodopseudomonas*, and *Polaromonas* in both metagenome communities, suggesting a synergistic degradation of benzoate. | Edited as per reviewer comment |
| Lines 413-419: | Kindly revise the sentences as follows;  On the other hand, genes encoding biphenyl 2,3 diol dioxygenase, benzaldehyde dehydrogenase, nap/bph dioxygenase, ortho-halobenzoate 1,2-dioxygenase, phenol dioxygenase, and 1,2-dihydroxycyclohexa-3,5-diene-1-carboxylate dehydrogenate were abundant in MGB-3 (Fig S5). Similarly, a two-way comparison between MGB-2 and MGB-3 of functional gene annotation using SEED subsystem for aromatic degradation revealed a significant level of abundance for benzoate ligase (p = 2.63 e-3) and 4-hydroxyphenylacetate 3-monooxygenase (p = 8.93 e-3) in MGB-2. | Revised as per reviewer comment |
| Lines 423-426: | Please amend the sentences as shown below;  Furthermore, a comparison between MGB-2 and MGB-3 at functional gene annotation using RefSeq for aromatic degradation revealed a significant level of abundance of *Acidothermus* (p=2.68 e-3), *Nakamurella* (p=6.39 e-3) in MGB-2 while *Mycobacterium* (p =3.30 e-3), *Leptothrix* (p=7.89 e-3) and *Polynuclearbacter* (p=0.034) in MGB-3 (Fig. S9). | Amended as per reviewer comment |
| Lines 430-432: | Kindly modify the sentences as observed below;  Therefore, the reconstructions of complete biphenyl/PCB and PAH degradative pathways, based on the annotated genes, were done and shown in Fig 6. Cytoscape-based networking revealed microbial interaction in the xenobiotic biodegradation pathway. | Modified as per reviewer comment |
| ***Figure Captions*** | | |
| Lines 665-667: | Please edit the caption as observed below; Fig 3 (A). Taxonomic analysis beta diversity of MGB-2 and MGB-3 on Bary -Curtis dissimilarity. (B) Krona plot demonstrated the relative abundance of bacterial taxa across phylum to genus level hierarchy of a. MGB-2; b. MGB-3 | Edited as per reviewer comment |
| ***Supplementary Information Document*** | | |
| Kindly edit the word “Supplementary” in all areas mentioned incorrectly throughout the Supplementary Information document. | Kindly amend the quoted caption as seen below;  Supplementary Fig S3. Rarefaction curve represented species richness indicating function of number of reads (y-axis) per MGB-2 and MGB-3 (x-axis) | Amended as per reviewer comment |
|  | Please edit the quoted caption as follows;  Supplementary Fig S5. Comparison between MGB-2 and MGB-3 of functional gene annotation using STAMP using SEED subsystem a.) Tol and Ton b.) two-component system c.) membrane transport d.) Phosphate transporter | Edited as per reviewer comment |
